# Context-dependent effects of formic acid on olfactory learning and generalisation in ants

**DOI:** 10.1038/s41598-025-10996-x

**Published:** 2025-07-10

**Authors:** Natacha Rossi, Martin Giurfa, Patrizia d’Ettorre

**Affiliations:** 1 Ecology and Evolution, School of Life Sciences, University of Sussex, Brighton, UK; 2 Sorbonne University, CNRS, Inserm, Neuro-SU, Paris, F-75005 France; 3 Sorbonne University, CNRS, Inserm, Institut of Biology Paris-Seine, IBPS, Paris, F75005 France; 4 Laboratory of Experimental and Comparative Ethology, UR4443, University Sorbonne Paris Nord, 99 avenue J.-B. Clément, Villetaneuse, France

**Keywords:** Chemical communication, Alarm pheromones, Associative learning, Insect neurobiology, Stimulus discrimination, Classical conditioning, Behavioural ecology, Chemical ecology

## Abstract

Animals use learning and memory to recognise cues that predict rewards or punishments, allowing flexible decision-making. When facing new stimuli, they often generalise – responding similarly to different but related cues – enabling adaptive behaviour despite natural variation. Pheromones, chemical signals central to social interactions, are known to affect learning and memory, but their role in generalisation is unknown. This study explores how formic acid, an alarm pheromone in *Camponotus aethiops* ants, influences odour discrimination and generalisation in an appetitive olfactory discrimination learning task. Using controlled conditioning, we found that formic acid affected learning asymmetrically: it enhanced discrimination when octanal was rewarded and hexanal punished but not when hexanal was rewarded and octanal punished, suggesting a shift in learning priorities. Unexpectedly, formic acid also increased responses to conditioned stimuli and novel odours but only when octanal was the rewarded stimulus. These findings suggest that formic acid modulates associative learning and generalisation in a context-dependent way. Rather than acting solely as an arousal trigger, formic acid appears to reshape cognitive processing by altering stimulus discrimination and odour sensitivity. This highlights a novel role for alarm pheromones in modulating cognition, with broader implications for understanding chemical communication in social insects and beyond.

## Introduction

To successfully navigate their environment, animals must differentiate between rewarding and punishing stimuli, forming associations that enable adaptive decision-making and enhance survival. Learning and memory allow animals to develop stable expectations about the consequences of specific stimuli, shaping their behavioural responses accordingly. When facing a new stimulus, animals can rely on generalisation to transfer learned associations to similar but novel stimuli^1–3^. This process is particularly relevant in dynamic environments, where sensory cues fluctuate over time and space^4^requiring flexible yet accurate responses.

In olfactory learning, animals may generalise responses based on chemical similarity, with stronger generalisation occurring when odours share functional groups or carbon-chain lengths^1,5,6^. While learning shapes generalisation patterns^2,5^external modulators – such as pheromones – could also influence this process. Given their role in modulating learning^7,8^pheromones may impact how animals generalise learned associations to novel stimuli. However, this possibility remains unexplored. Pheromones have been defined as chemical signals that facilitate intraspecific communication, eliciting stereotyped behavioural and physiological responses in conspecifics^9^. While pheromones are well known for coordinating social interactions, recent research suggests they also modulate cognitive processes such as learning and memory, influencing how insects perceive and respond to olfactory stimuli. Studies have shown that pheromones can affect learning across various insect taxa. For instance, in honeybees, exposure to alarm pheromones has been found to impair appetitive learning, whereas appetitive pheromones enhance it^10–12^.

Ants are ideal model organisms for studying the effects of pheromones on associative learning due to their reliance on chemical communication and sophisticated odour-based behaviours^13,14^. Recently, studies have attempted to investigate pheromone influences on associative learning using free-walking ant paradigms. However, these experiments have yielded negative results, particularly regarding the effects of appetitive pheromones on appetitive learning^15–17^. These findings suggest that pheromone effects on learning may be context-dependent or constrained by methodological limitations in free-walking setups. Given these challenges, an alternative approach with greater experimental control is necessary. *Camponotus aethiops* ants serve as an excellent model for investigating alarm pheromone modulation of learning for several reasons. First, their alarm pheromone has been identified as formic acid, allowing for precise experimental manipulation of its effects. Formic acid is a key defensive and alarm compound in formicine ants, released from the venom gland in response to threats, where it acts as both a repellent and a chemical cue triggering collective defensive behaviours^18^. Second, *C. aethiops* is a well-established model species for studying appetitive learning, particularly using the *maxilla-labium* extension response (MaLER) paradigm, which enables conditioning of an odour (conditioned stimulus or CS) paired with sucrose reward (unconditioned stimulus or US) and thus controlled assessments of olfactory learning and memory^5,19^. Finally, prior research has demonstrated that formic acid enhances discrimination between nestmate and non-nestmate cuticular profiles in this species^20,21^suggesting its potential role in modulating broader cognitive processes such as associative learning and generalisation.

In this study, we investigated whether formic acid, the primary alarm pheromone in *C. aethiops*, influences both differential odour learning and generalisation. Using the MaLER paradigm with restrained ants, we were able to precisely control stimulus presentation and response measurement, ensuring a rigorous assessment of learning processes. Based on the findings that formic acid enhances discrimination between nestmate and non-nestmate cuticular profiles in *C. aethiops*^20,21^we hypothesised that formic acid exposure would enhance discrimination between a rewarded (CS+) and a punished (CS-) conditioned odorant. Given that increased discrimination is typically linked to reduced generalisation^2^we predicted that formic acid-exposed ants would show lower responses to novel odours compared to controls, supporting the idea that alarm pheromones sharpen associative learning by modulating stimulus differentiation.

## Materials and methods

### Study Organism

Experiments were conducted from March to May 2018, at the Laboratory of Experimental and Compared Ethology, Villetaneuse, France. We used five queen-right colonies of *C. aethiops* collected in 2014 and 2016 at Pompertuzat (Midi-Pyrénées, France, latitude 43.5, longitude 1.5167) and kept in the laboratory under controlled conditions (25 °C, light-dark cycle = 12:12, 36% humidity) in two Fluon©-coated plastic boxes connected by a tube. One box was provided with plaster floor and covered by cardboard (nest), the other was exposed to light and had sand on the floor (foraging area). Ants were fed twice a week with a mixture of honey and apples for carbohydrates and vitamins, and with pieces of crickets for proteins; water was provided *ad libitum.* Two weeks prior to the onset of the experiments, the ants’ diet was changed to crickets and water *ad libitum* but no carbohydrates were provided to increase the insects’ motivation for sucrose used as reward during conditioning.

### Conditioning and test procedures

***(a) Individual handling***.

Medium-sized workers were collected because they usually forage for food^22^. They were subsequently anaesthetized on ice and individually harnessed, following the procedure described by Perez et al. (2015). Afterward, they were placed in a dark, humid box for 3 h to recover from anaesthesia and acclimate to the harnessing conditions.

***(b) Pheromone exposure***.

Formic acid (Sigma-Aldrich, France) was diluted to 12% (3 µl pheromone + 22 µl water), equivalent to one third of the content of a single poison gland^18^. Ants were assigned to either the experimental group, exposed to formic acid, or the control group, exposed to 25 µL of pure water. Harnessed ants were individually confined for 15 min in a 50 ml plastic flask containing a filter paper (1 × 5 cm) soaked with either the pheromone solution or water, placed under a fume hood. After exposure, ants were directly transferred to a different fume hood for conditioning.

***(c) Stimuli***.

Ants were subjected to a differential conditioning in which an appetitive 1.80 M sucrose solution and an aversive 3 M NaCl solution (purity 99.5%, Sigma Aldrich, France) were used as reinforcements. Ants were trained to discriminate between two floral odours, octanal and hexanal (Sigma Aldrich, France), which differ by two carbons in their chain length. These odorants served as conditioned stimuli (CS), with one paired with sucrose (CS+), and the other paired with NaCl (CS-). Before each training phase, two microliters of pure odorant were applied to a 1 cm^2^ piece of filter paper, which was then inserted in a 10 ml plastic syringe for odour delivery. During memory tests, besides assessing responses to the CS + and the CS- in the absence of reinforcement, we also evaluated generalisation towards novel odorants, and the impact of formic acid on this process. Five aldehydes were used that varied along the carbon-chain length gradient. Two were the trained odorants (hexanal and octanal) while the three others (heptanal, nonanal and decanal) served as novel stimuli.

***(d) Conditioning and test procedures***.

To test whether olfactory generalisation gradients were influenced by pheromone exposure, ants were exposed to pheromone or water (control) and then subjected to differential conditioning of the MaLER, an appetitive reaction to sucrose stimulation^24^. Separate groups of ants were trained to discriminate two odorant combinations: octanal+/hexanal-, and hexanal+/octanal-; where ‘+’ indicates the presence of reward and ‘-’ that of punishment. Eight replicates of 12 ants were performed for each subgroup within an odour pair (e.g., octanal+/hexanal-, and hexanal+/octanal-), leading to a total of 192 ants (96 exposed to formic acid and 96 exposed to water).

Training consisted of 12 trials (six reinforced and six punished) during which the two CSs were presented in a pseudo-random sequence (e.g. ABABBABAABAB). The same stimulus was never presented more than twice consecutively. For half of the individuals of a group, the sequence started with the CS + while for the other half, it started with the CS-. Each trial lasted 1 min. Twenty-five seconds after placing the ant under a binocular microscope, a CS was presented during 5 s by delivering an air puff from a syringe positioned 2 cm from the ant’s head. Three seconds after the onset of odour presentation, the ant’s *maxilla-labium* was stimulated during 5 s with sucrose in the CS + trials, while NaCl was used in the CS- trials. Thus, the overlap between odour and reinforcement was always 2 s. To prevent a predictive, forward association between context and reinforcement, the ant remained in the conditioning place for an additional 27 s. The mechanical stimulation of the air puff was common to both CS + and CS- trials so that it could not act as predictor of appetitive reinforcement. The entire protocol was conducted under a fume hood to remove residual odour stimulations. The inter-trial interval was 12 min. Individuals that did not respond at least three times to the sucrose reward were discarded^23^ (9 out of 192 ants, 4.69% in total), to prevent confounding effects of a low motivation for the appetitive US on acquisition rates of the CS+.

During the unrewarded memory test, ants were presented with the five aldehydes in a randomised order ten minutes after the last conditioning trial. As in the training phase, each test lasted 1 min with odours presented for 5 s without reinforcement. The inter-test interval was 12 min. After the end of the test phase (all five odorants tested, ca. 1 h), a droplet of sucrose was presented to each ant to verify the presence of MaLER to the appetitive US. Seven out of 192 ants (3,65%) for which measurements could not be obtained were discarded from analyses; the mortality rate during the experiment was of 1.04% (2 out of 192 ants). The number of ants retained in the analyses were 89 for the octanal+/hexanal- condition (46 formic-acid exposed ants and 43 control ants), and 85 for the hexanal+/octanal- condition (42 formic-acid exposed ants and 43 control ants).

### Odour pairing schedule and colony representation

The two odour pairings were tested at different times: the octanal⁺/hexanal⁻ pairing was conducted in March–April 2018, and the hexanal⁺/octanal⁻ pairing in May 2018. Thus, odour pairing was not randomised over calendar time, and each occurred on separate dates. While most colonies contributed ants to both odour pairings, representation was not fully balanced due to variation in forager availability across colonies and time. In contrast, representation across treatment groups (formic acid vs. water) was well balanced, with ants from each colony typically distributed across both treatments. To account for colony-specific variation in learning or responsiveness, colony identity was included as a random effect in all statistical models.

#### Data analysis

All statistical analyses were conducted in R (version 4.4.2^25^) and the significance threshold was set at α = 0.05. Generalised linear mixed models (GLMMs) were used to analyse the probability of the MaLER as a function of trial number, conditioned stimulus, odour identity, odour pairing, and treatment condition. Models were fitted using the glmmTMB package^26^and significance was assessed using Type III Wald chi-square tests with the car package^27^. All GLMM assumptions were checked by visualising residual distributions and fitted values using the DHARMa package^28^. Plots were obtained using the package ggplot2 ^29^.

To assess learning, a GLMM with a binomial error distribution and a logit link function was fitted, including trial number (1–6), conditioned stimulus (CS⁺ or CS⁻), odour pairing (octanal⁺/hexanal⁻ or hexanal⁺/octanal⁻), and treatment (formic acid or water) as fixed effects and MaLER (0/1) as the response variable. Two-way interaction terms (trial × CS, trial × odour pairing, CS × odour pairing, and treatment x odour pairing) were included to evaluate whether learning patterns differed depending on the conditioned odours and treatment conditions. Colony ID and individual ant ID (nested within colony) were included as random effects to account for repeated measurements, inter-individual and inter-colony variation.

To assess memory retention and generalisation, a second GLMM was fitted using the post-conditioning test data, with MaLER (0/1) as the response variable. Fixed effects included odour identity (hexanal, heptanal, octanal, nonanal, decanal), conditioned odour pairing (octanal⁺/hexanal⁻ or hexanal⁺/octanal⁻), and treatment (formic acid or water), along with two-way interaction terms (odour identity × odour pairing and treatment × odour pairing). Colony ID and individual ID (nested within colony) were included as random effects to account for inter-individual and inter-colony variation.

Post-hoc comparisons were performed using estimated marginal means (emmeans) to assess specific differences in MaLER responses between odours within each conditioned odour pairing and treatment condition^30^. P-values were adjusted for multiple comparisons using Tukey’s method.

A Bayesian multinomial logistic regression model was fitted to assess the effects of trial, treatment, and odour pairing on the ants’ discrimination responses (Δ = MaLER(CS⁺) − MaLER(CS⁻)). The model included random effects for colony ID and ant ID (nested within colony) to account for inter-individual and inter-colony variation.

Posterior predictive checks indicated a good model fit, with the Bayesian p-value for standard deviation (*p* = 0.30) confirming that the model accurately captured the variability in the data. All Rhat values were between 1.00 and 1.02, indicating good convergence. Effective sample sizes (ESS) were sufficient for reliable posterior estimates (Bulk ESS > 1000, Tail ESS > 1500 for most parameters).

To further examine response specificity, we computed a discrimination-based specificity index for each ant, defined as the difference between MaLER responses to the CS⁺ and CS⁻, adjusted by the maximum response to any novel odour. This index was treated as an ordinal response variable and analysed using a cumulative link mixed model (CLMM) with treatment and odour pairing as fixed effects and colony ID as a random intercept. The model was fitted using the ordinal package^31^ with a logit link function and the Laplace approximation for random effects. Model fit and convergence were assessed based on gradient size, number of iterations, and condition number, and were deemed acceptable.

## Results

### Learning

**Fig. 1:**
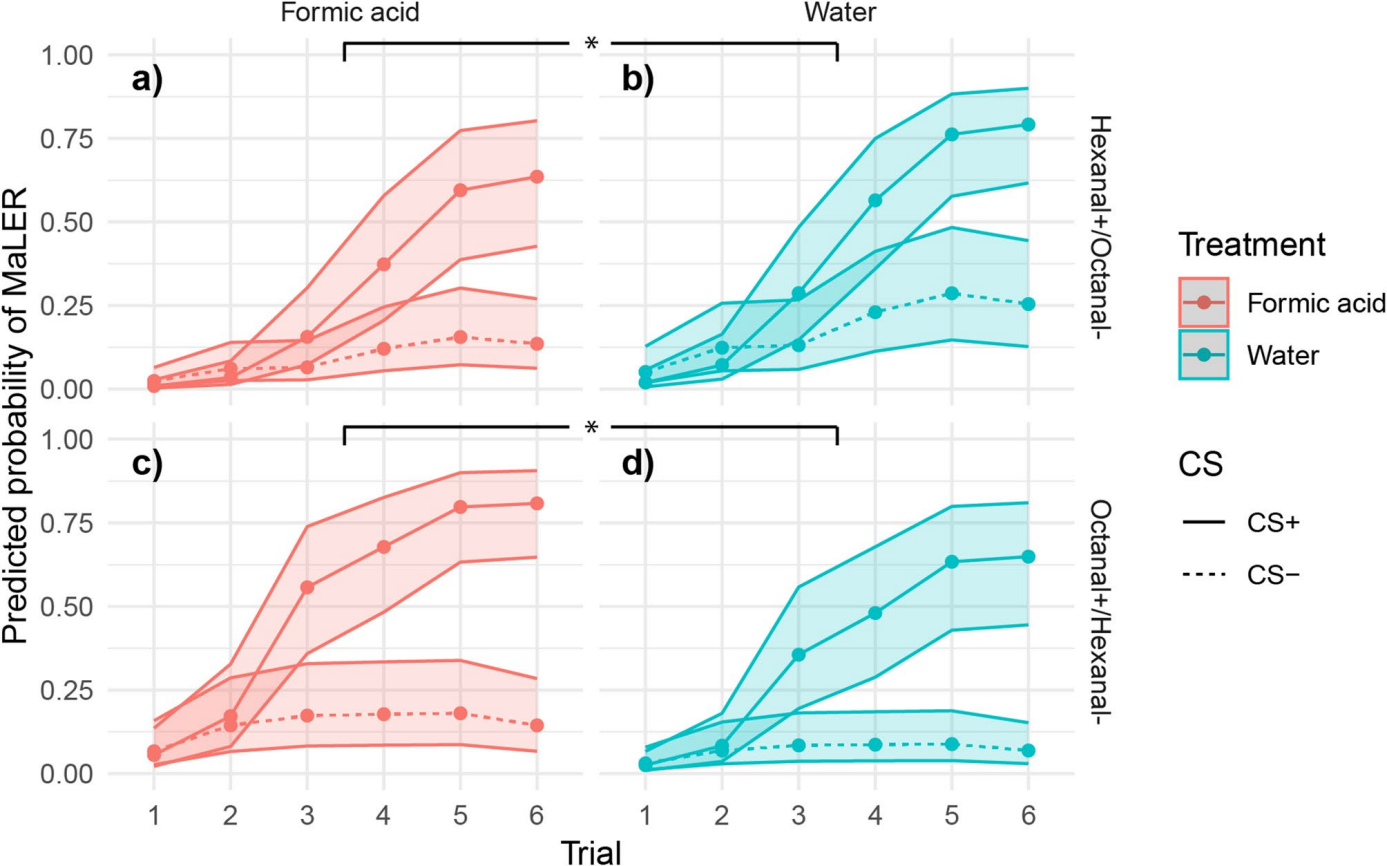
**Predicted probability of MaLER across trials as a function of conditioned stimulus** (CS^+^/CS^-^),** treatment**,** and odour pairings.** The x-axis represents the trial number, while the y-axis shows the predicted probability of MaLER (0–1). Solid lines represent CS⁺ (sucrose-rewarded odour), and dotted lines represent CS⁻ (salt-punished odour). Treatment conditions (formic acid vs. water) are colour-coded, with shaded areas indicating 95% confidence intervals. Panels are faceted by odour pairings (rows) and treatment (columns) to illustrate the interactive effects of odour, treatment, and trial progression on MaLER responses. Panel (**a**) predicted probability of MaLER to hexanal as CS + and octanal as CS- for ants pre-exposed to formic acid. Panel (**b**) predicted probability of MaLER to hexanal as CS + and octanal as CS- for ants pre-exposed to water. Panel (**c**) predicted probability of MaLER to octanal as CS + and hexanal as CS- for ants pre-exposed to formic acid. Panel (**d**) predicted probability of MaLER to octanal as CS + and hexanal as CS- for ants pre-exposed to water. Predictions were obtained from the generalised linear mixed model (GLMM), accounting for random effects of colony and individual identity. Formic acid inhibited MaLER in the hexanal+/octanal- condition but enhanced it in the octanal+/hexanal- condition compared to water. GLMM, treatment × odour pairing: * *p* < 0.05.

Overall, ants successfully learned to discriminate the CS⁺ from the CS⁻, as indicated by a significant trial × CS interaction (χ² = 79.25, df = 5, *p* < 0.001; Fig. 1). From trial 3 onwards, MaLER probabilities were significantly higher for CS⁺ than for CS⁻ (*p* < 0.001), demonstrating a progressive learning effect.

A significant CS × odour pairing interaction (χ² = 9.77, df = 1, *p* = 0.002) indicated that learning differed between odour pairings. The contrast between hexanal and octanal for the CS + revealed a significant difference, with hexanal being associated with a lower likelihood of MaLER to the CS⁺ compared to octanal (OR = 0.523, SE = 0.164, z = −2.071, *p* = 0.038). However, in the CS- condition, the contrast between hexanal and octanal did not yield a significant effect (OR = 1.176, SE = 0.378, z = 0.503, *p* = 0.615), indicating no meaningful difference in MaLER likelihood between these odorants when associated with CS-. There was also a significant main effect of treatment (χ² = 4.36, df = 1, *p* = 0.037), indicating that formic acid exposure influenced overall MaLER response probabilities during learning.

Additionally, there was a treatment × odour pairing interaction (χ² = 9.37, df = 1, *p* = 0.002), showing that the effect of formic acid treatment depended on the conditioned stimuli. For the hexanal+/octanal- pairing, the results indicated that exposure to formic acid (Fig. 1a) significantly reduced the odds of this stimulus pair being associated with MaLER compared to water (Fig. 1b) (OR = 0.459, SE = 0.171, z = −2.088, *p* = 0.037). This suggests that formic acid may have an inhibitory effect on learning when hexanal was the reinforced odour.

Conversely, for the octanal+/hexanal- pairing, exposure to formic acid (Fig. 1c) significantly increased the odds of this stimulus pair being associated with MaLER compared to water (Fig. 1d) (OR = 2.280, SE = 0.833, z = 2.255, *p* = 0.024). This indicates that formic acid may enhance learning when octanal was the reinforced odour.

These results highlight a context-dependent effect of formic acid on olfactory learning, suggesting that formic acid does not uniformly influence conditioned responses but instead interacts with specific odour pairings in a differential manner.

**Fig. 2:**
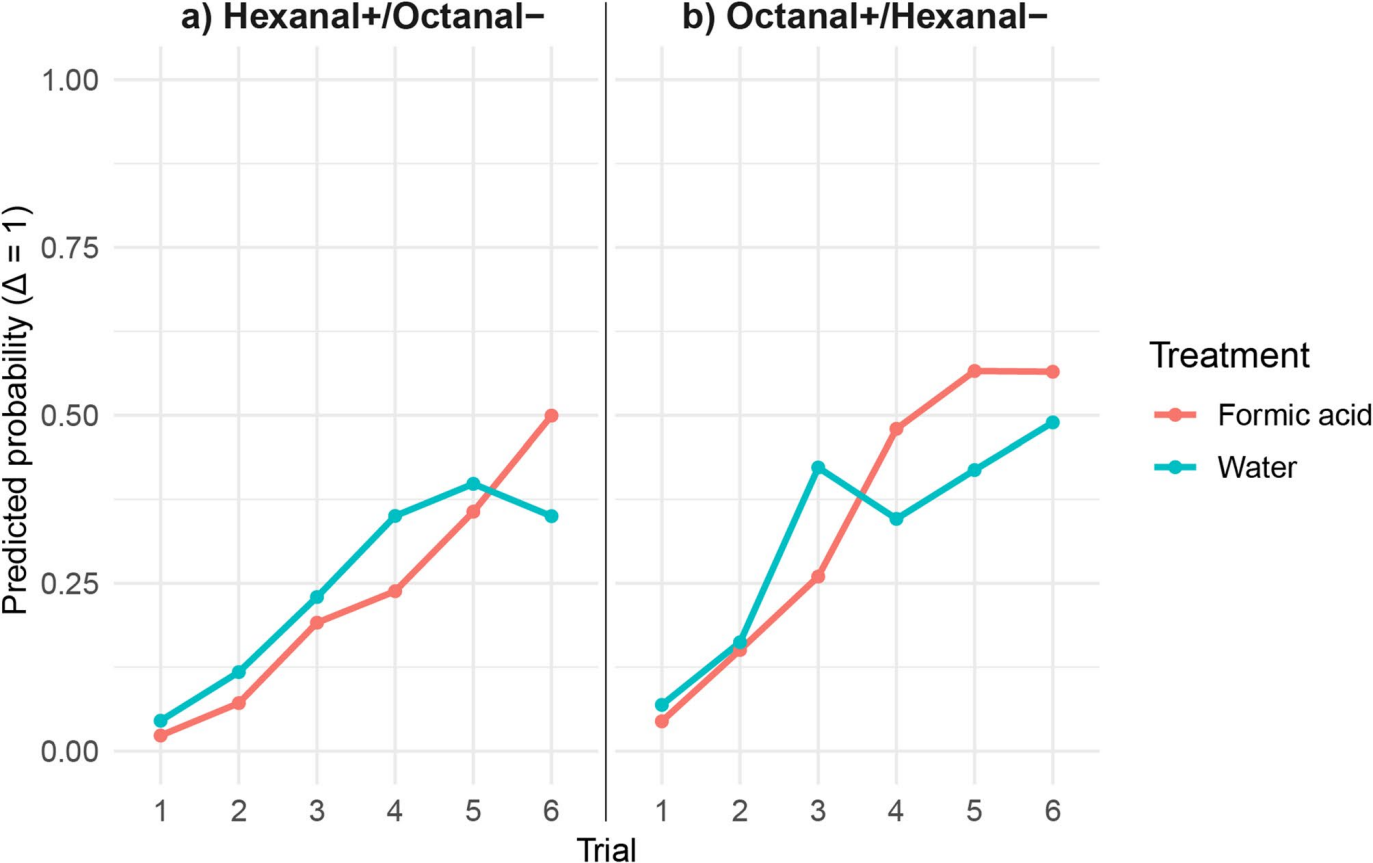
Predicted probability of discrimination responses (Δ = 1) across conditioning trials in ants exposed to either formic acid (red) or water (blue). The probability of discrimination was modelled using a Bayesian multinomial regression, accounting for the effects of trial (learning progression), treatment (pheromone pre-exposure), and odour pairing. Panels represent different odour pairings: hexanal+/octanal− (**a**) and octanal+/hexanal− (**b**). Lines indicate the mean predicted probability for each treatment group, with points representing individual trials. Formic acid-exposed ants showed higher discrimination performance in the octanal+/hexanal- condition compared to the hexanal+/octanal- condition.

To further quantify discrimination learning, we computed Δ = MaLER (CS⁺) - MaLER (CS⁻) and modelled it as an ordered categorical variable in a Bayesian multinomial logistic regression. A significant three-way interaction between trial, treatment, and odour pairing (β = −33.36, 95% CI [−121.32, −3.04]) revealed that the effect of formic acid pre-exposure on discrimination responses changed across trials and depended on the specific conditioned odours (hexanal+/octanal- or octanal+/hexanal-) (Fig. 2).

Post hoc contrasts were performed to compare discrimination responses between water-exposed and formic acid-exposed ants, separately for the hexanal+/octanal − and octanal+/hexanal − conditions. In the hexanal+/octanal − condition (Fig. 2a), formic acid-exposed ants showed a higher discrimination response in trial 6 compared to water-exposed ants, but this difference was not statistically supported (95% CI: [−0.062, 0.387]). In earlier trials, discrimination differences between treatment groups were also not statistically significant (Trial 4: 95% CI: [−0.347, 0.086]; Trial 5: 95% CI: [−0.251, 0.175]).

In the octanal+/hexanal − condition (Fig. 2b), formic acid pre-exposure did not significantly alter discrimination in early trials (1–3), but in trials 4 and 5, ants pre-exposed to formic acid showed higher discrimination compared to water-exposed ants. However, these differences were not statistically significant (Trial 4: 95% CI: [−0.074, 0.354]; Trial 5: 95% CI: [−0.068, 0.383]). In trial 6, there was no strong evidence of a difference between the two groups (95% CI: [−0.144, 0.283]), probably indicating that maximum discrimination was reached at the end of the conditioning and that formic acid could not increase it further.

To further investigate the role of conditioned stimulus identity, post hoc contrasts were performed to assess differences in discrimination between the octanal+/hexanal- condition vs. the hexanal+/octanal- condition, separately for water-exposed and formic acid-exposed ants. These contrasts revealed that in the water-exposed group, there was no significant difference in discrimination between the octanal+/hexanal- and hexanal+/octanal- conditions across trials (Trial 1: 95% CI: [−0.204, 0.072]; Trial 3: 95% CI: [−0.475, 0.002]; Trial 6: 95% CI: [−0.405, 0.031]). These results indicate that, in the absence of formic acid pre-exposure, ants did not exhibit a clear preference for discriminating one combination of odours over the other.

In contrast, among formic acid-exposed ants, discrimination responses differed significantly between the octanal+/hexanal- and hexanal+/octanal- conditions in trials 4 and 5. Specifically, when formic acid-exposed ants were conditioned with octanal+/hexanal-, their discrimination was significantly higher than when conditioned with hexanal+/octanal- (Trial 4: 95% CI: [−0.514, −0.029]; Trial 5: 95% CI: [−0.464, −0.013]). These results suggest that formic acid pre-exposure selectively enhanced discrimination in the octanal+/hexanal- condition. However, in trial 6, this difference was no longer significant (95% CI: [−0.327, 0.116]).

Overall, these results suggest that formic acid pre-exposure may modulate odour discrimination in ants, but its effect is not uniform across trials and depends on the conditioned stimulus identity.

### Memory and generalisation

**Fig. 3:**
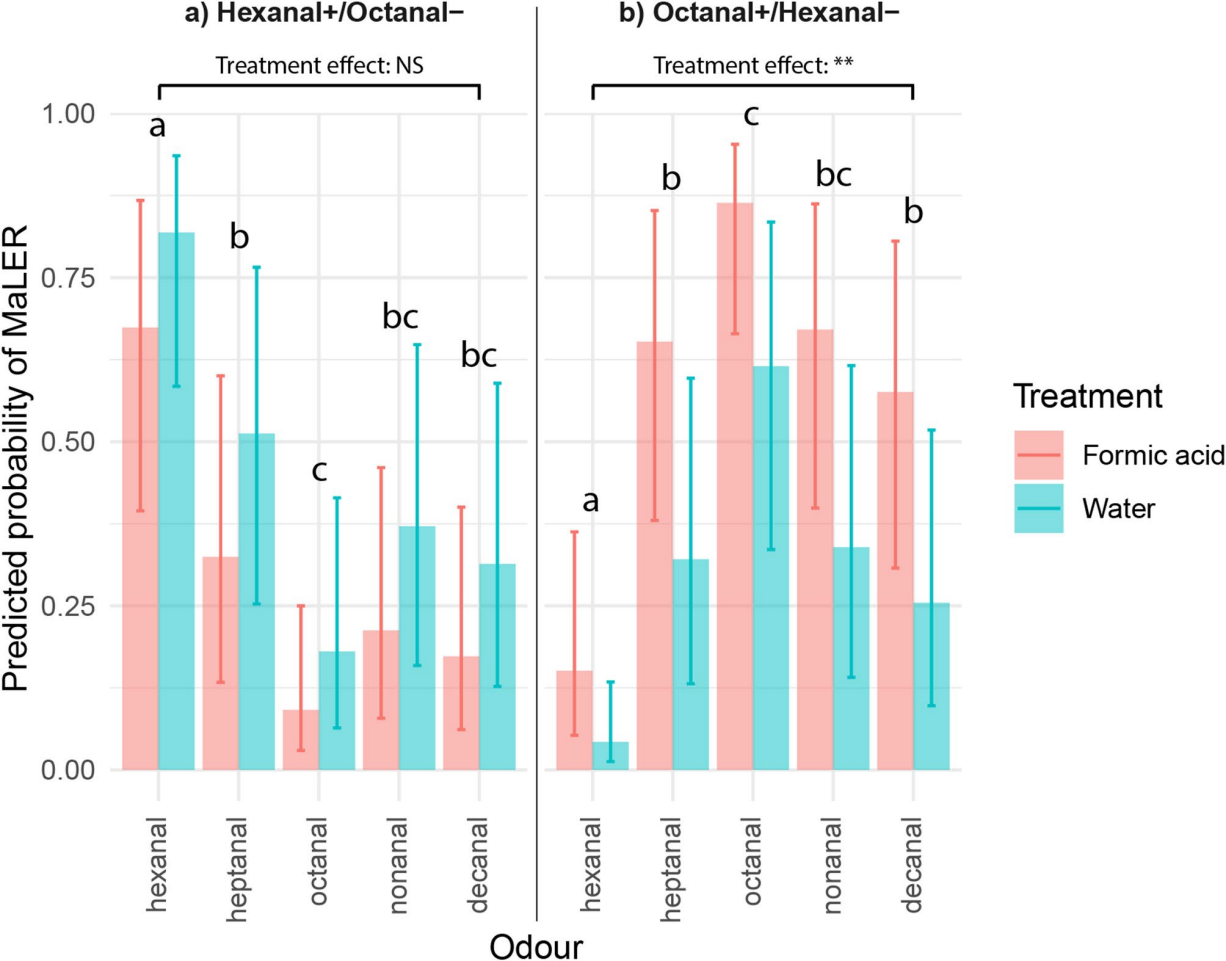
Predicted probability of MaLER in ants exposed to different odours following conditioning with either hexanal as CS + and octanal as CS- (**a**) or octanal as CS⁺ and hexanal as CS- (**b**). Bars represent the predicted probabilities of MaLER (Mean ± 95% CI) for each odour, separated by odour pairing (facet panels) and treatment (formic acid vs. water, indicated by bar fill). Predictions were obtained from the generalised linear mixed model (GLMM), accounting for random effects of colony and individual identity. Responses varied depending on the odour tested, the odour pairing used during conditioning, and the treatment condition, with formic acid significantly increasing response probability only when octanal was CS⁺. Letters above bars indicate statistical groupings based on Tukey-adjusted comparisons: bars sharing a letter do not differ significantly (*p* > 0.05). Horizontal brackets above each panel indicate the effect of treatment for each odour pairing: **NS** = not significant; ** = *p* < 0.01.

A significant odour identity × odour pairing interaction (χ² = 86.96, df = 4, *p* < 0.001) indicated that responses to the five odours (two conditioned stimuli and three novel odours) during the post-conditioning tests depended on the pair of odours used during conditioning (Fig. 3).

When hexanal was the CS⁺ and octanal the CS⁻ (Fig. 3a), responses to hexanal were significantly higher than to octanal (*p* < 0.001), confirming successful discrimination and retention of learning. Responses to heptanal were significantly lower than to hexanal (*p* = 0.006) but significantly higher than to octanal (*p* = 0.003), indicating partial generalisation from the CS + while maintaining discrimination from CS⁻. In contrast, responses to nonanal and decanal were significantly lower than to hexanal (*p* < 0.001) and not significantly different from octanal (*p* > 0.05), suggesting that these odours were perceived similarly to CS⁻ rather than as intermediate between CS⁺ and CS⁻.

These results highlight the influence of carbon chain length on odour perception. A one-carbon increase from hexanal (C6) to heptanal (C7) led to partial generalisation, while a two-carbon difference (hexanal to octanal, C8) allowed clear discrimination. Longer-chain aldehydes (nonanal, C9; decanal, C10) were perceived more like octanal compared to hexanal, suggesting a categorical shift in odour quality.

When octanal served as the CS⁺ and hexanal as the CS⁻ (Fig. 3b), responses to octanal were significantly higher than to hexanal (*p* < 0.001), confirming both stimulus discrimination and learning retention. Responses to heptanal were also significantly higher than to hexanal (*p* < 0.001), but significantly lower than to octanal (*p* = 0.037), indicating partial generalisation from the CS⁺ while maintaining discrimination. Decanal responses were significantly lower than to octanal (*p* = 0.003) but higher than to hexanal (*p* < 0.001), suggesting an intermediate percept. Responses to nonanal were significantly higher than to hexanal (*p* < 0.001) but not significantly different from octanal (*p* = 0.062), indicating partial generalisation with less clear discrimination.

The one-carbon difference between octanal (C8) and heptanal (C7) led to partial generalisation, whereas the two-carbon gap between octanal and hexanal (C6) resulted in clear discrimination. In contrast to the hexanal⁺/octanal⁻ condition, responses to decanal (C10) in this condition were significantly higher than to hexanal and lower than to octanal. Nonanal (C9), differing by only one carbon from octanal, elicited responses similar to those for the CS⁺, while decanal (C10), with two additional carbons, reflected a gradual shift in perceived odour quality with increasing chain length.

A significant treatment × odour pairing interaction (χ² = 8.62, df = 1, *p* = 0.003) revealed that treatment effects varied across odour pairings. In the hexanal+/octanal- condition, formic acid treatment did not significantly alter response probabilities across odours (*p* = 0.131). However, in the octanal⁺/hexanal- condition, ants treated with formic acid exhibited significantly higher response probabilities than those treated with water (*p* = 0.007), indicating that formic acid enhanced overall responsiveness in this condition.

These results demonstrate that ants generalise their conditioned response along a structured molecular gradient, with responses decreasing as the carbon chain difference from CS⁺ increases. However, generalisation patterns were asymmetrical, as decanal was grouped with CS⁻ when hexanal was CS⁺ but treated as intermediate when octanal was CS⁺. Additionally, formic acid modulated response strength only in the octanal+/hexanal- condition, suggesting that its effects depend on the specific odour pairing.

To further examine response specificity, we calculated an index reflecting each ant’s ability to discriminate between the CS⁺ and CS⁻ while limiting generalisation to novel odours. The distribution of index values was centred near zero and showed high individual variability, with some ants displaying strong specificity and others responding more broadly or inconsistently. Statistical analysis revealed no significant effect of treatment (*z* = 0.39, *p* = 0.694), odour pairing (*z* = − 0.66, *p* = 0.507), or their interaction (*z* = 0.95, *p* = 0.342), indicating that formic acid did not reliably influence the specificity of conditioned responses.

## Discussion

This study provides new evidence that alarm pheromones released by ants do more than trigger spontaneous defensive behaviours – they actively shape associative learning in these insects. By investigating the effects of formic acid – a substance characterised as an alarm pheromone in Formicine ants – on olfactory discrimination and generalisation in *Camponotus aethiops*, we reveal that its influence is context-dependent, challenging the traditional view that alarm pheromones function exclusively as arousal signals. We also evaluated the impact of this pheromone on odour generalisation following differential conditioning. Our study tested the hypothesis that formic acid exposure enhances discrimination between conditioned stimuli (CS⁺ and CS⁻) and reduces generalisation to novel odours. While our results confirm that formic acid modulates appetitive differential olfactory learning and odour responsiveness, its effects varied depending on the specific conditioned odours used. Such an effect could be adaptive in nature by allowing ants to fine-tune their responses to ecologically relevant cues, ensuring more efficient foraging and accurate threat assessment.

Two main results emerged from our work: (1) formic acid significantly altered the acquisition dynamics of appetitive differential conditioning of the MaLER. Specifically, ants exposed to formic acid showed improved discrimination when octanal was rewarded and hexanal punished compared to when hexanal was rewarded and octanal punished, suggesting that formic acid modifies how odour information is processed during learning. (2) In the retention and generalisation tests, formic acid exposure led to an increase in overall response levels to both the conditioned stimuli (CS⁺ and CS⁻) and the novel odours compared to water, but only when octanal was rewarded and hexanal punished. This general increase suggests that formic acid modulated olfactory responsiveness more broadly, rather than enhancing responses selectively to the conditioned stimuli. This result only partially supports our hypothesis, as discrimination between the CS⁺ and CS⁻ was enhanced by formic acid during the learning phase, but this effect was observed exclusively in the octanal⁺/hexanal⁻ condition. In contrast, formic acid did not sharpen discrimination or reduce generalisation in the post-conditioning test, as might be expected if it enhanced stimulus specificity. Rather, it increased responsiveness to novel odours in the octanal⁺/hexanal⁻ condition, indicating that its effect was more consistent with a global increase in olfactory sensitivity than with a refinement of associative generalisation. This dual effect – heightened responsiveness alongside altered learning dynamics – suggests a more complex interaction between alarm pheromones and olfactory cognition than previously assumed.

It is also worth noting that our differential conditioning paradigm involved an aversive CS⁻ (NaCl), rather than a neutral, non-reinforced odour. This design introduces the possibility that ants formed both positive and negative odour associations, which may influence how novel odours are evaluated. However, our results do not support the idea that formic acid alters generalisation through increased aversive generalisation from the CS⁻: we observed increased, not reduced, responses to novel odours in the octanal⁺/hexanal⁻ condition and no effect of treatment in the hexanal+/octanal- condition. Thus, while dual reinforcement could influence generalisation patterns in principle, the effect of formic acid in our experiment is better explained by an overall increase in olfactory responsiveness in certain contexts rather than by changes in the generalisation due to punishment. Future studies using a broader set of novel odours that vary systematically on either side of the conditioned stimuli could help disentangle whether ants generalise based on similarity to CS⁺, CS⁻, or both.

Our results show that formic acid exposure significantly altered the acquisition dynamics of differential olfactory conditioning. Ants exposed to formic acid exhibited improved discrimination when octanal was rewarded and hexanal punished compared to when hexanal was rewarded and octanal punished. This suggests that formic acid selectively modulates how odour information is processed, rather than having a uniform enhancing or impairing effect on learning.

Discrimination performance was dependent on the intrinsic properties of the odours used. Although there was no significant difference in discrimination between the hexanal+/octanal- and octanal+/hexanal- conditions for control ants, their overall response in the former condition was higher than in the latter, a pattern already observed by Perez et al. (2016). Formic acid-exposed ants displayed the opposite pattern, favouring discrimination when octanal was rewarded and hexanal punished. This reversal suggests that formic acid may amplify attention to unexpected reward associations, increasing responsiveness when the less-preferred odour is reinforced. The observed interaction between odour preference, formic acid exposure, and learning performance highlights the role of hedonic biases in shaping learning under pheromone influence.

We propose that the lack of congruence between the alarm signal and the appetitive learning context contributes to the asymmetries observed in odour learning. This inconsistency between signal and learning valence has already been shown to induce negative effects of alarm pheromones on appetitive learning in honeybees^10–12^. Indeed, when honeybees are exposed to the alarm pheromone 2-heptanone, their appetitive learning is reduced compared to a control group exposed to mineral oil^11^.

Further supporting a context-dependent interpretation, previous research in honeybees has demonstrated that pheromone pre-exposure modulates behavioural responsiveness and learning in a valence-specific manner. For instance,^32^ showed that an appetitive pheromone (geraniol) reduced aversive responsiveness, while an aversive pheromone (isopentyl acetate) counteracted fatigue-related decreases in shock responsiveness. Similarly, Baracchi et al. (2017) found that aversive pheromones such as isopentyl acetate and 2-heptanone significantly reduced sucrose responsiveness, whereas the appetitive pheromone geraniol enhanced it. More recently, Baracchi et al. (2020) demonstrated that geraniol improved, and 2-heptanone impaired, olfactory learning and memory through durable changes in aminergic pathways regulating motivational state. Although these effects have not yet been investigated in ants, the asymmetrical effects observed in our study, modulated by both odour identity and reward contingency, suggest a similar form of pheromone-induced cognitive modulation that extends beyond simple non-associative pre-exposure effects.

However, if the incongruence between the alarm signal and learning context were the sole driver of the effects observed in our study, similar impairments in learning should also be evident for hexanal+/octanal- and octanal+/hexanal-, yet this does not appear to be the case in *Camponotus* ants. This suggests that additional mechanisms may be at play in shaping the observed asymmetries in odour learning.

Studies on hedonic values of sweeteners in aversive gustatory conditioning have shown that preference biases influence discrimination performance^33^. Therefore, we suggest that it would be easier to learn a discrimination with a non-preferred rewarded odour (octanal) in the presence of formic acid than with a preferred odour (hexanal), in which case the incoherence between the hedonic value of hexanal and the alarm signal would be higher. While our results suggest that the observed modulation of learning by formic acid stems from an inconsistency between the alarm signal and the learning context, this hypothesis remains to be formally tested.

Our results suggest that formic acid does not simply enhance or impair learning but instead shifts learning priorities in a context-dependent manner. One possible explanation is that formic acid increases attention to unexpected reward associations, making discrimination easier when the non-preferred odour is rewarded. In contrast, when the preferred odour is rewarded, this heightened vigilance may introduce interference, reducing discrimination. Such effects align with studies in other insects, where alarm pheromones alter learning by biasing individuals toward vigilance and avoidance strategies rather than reward-driven learning^10–12^. This could be explained by formic acid modulating odour salience at the neural level, potentially influencing how odours are processed in the antennal lobe and/or mushroom body.

Alternatively, alarm pheromones may induce a heightened state of arousal or vigilance, which could enhance sensitivity to odours in general while simultaneously influencing associative processing. This aligns with the hypothesis proposed by Rossi et al. (2019) that discrimination errors arise due to limited cue sampling and that alarm pheromones may lower perception thresholds, increasing the amount of information available to the individual. This increase in information would be associated with an increase in the probability of detecting differences between odours. In this context, formic acid might enable ants to process a broader range of olfactory cues, enhancing their ability to differentiate between stimuli. Despite differences in overall responsiveness, we found no significant differences in a specificity index comparing CS⁺, CS⁻, and novel odour responses. This suggests that formic acid did not sharpen generalisation gradients per se but instead modulated the intensity of olfactory responsiveness in a context-dependent manner. This is supported by the observed increase in responses to both the conditioned and novel odours during the memory and generalisation test compared to water, but only in the octanal⁺/hexanal⁻ condition. This suggests that formic acid exposure amplifies overall olfactory sensitivity in a context-dependent manner, rather than functioning exclusively to enhance discrimination processes. Given these findings, it is likely that both mechanisms – heightened general odour sensitivity and altered associative learning – interact to shape the observed behavioural responses. Future studies using neural imaging or pharmacological interventions could clarify whether formic acid alters neuromodulatory pathways, particularly those involving octopamine and dopamine, which are known to modulate appetitive responsiveness and reward processing^34,35^ and thus appetitive learning and attention^11,36^.

Given that formic acid is an alarm pheromone, it is possible that it interacts with specific glomerular subsets in the antennal lobe, altering the perception and discrimination of odours during learning. Mizunami et al.^36^ reported that in ants, alarm pheromones are processed through distinct neural circuits that interact with higher-order learning and memory centres, suggesting that formic acid may similarly engage a specialised pathway that modulates olfactory discrimination. Their findings indicate that alarm pheromones can enhance sensory responsiveness and affect neural plasticity in associative regions such as the mushroom body, potentially explaining why formic acid alters both learning and generalisation patterns in ants. This supports the hypothesis that formic acid does not merely increase arousal but actively reshapes how olfactory stimuli are processed and categorised during learning.

Our findings provide novel insights into how associative learning and olfactory generalisation operate in ants. We demonstrate that a pheromone modulates generalisation and learning asymmetrically in ants, challenging conventional views of how pheromones influence behaviour. Unlike previous research that has focused on pheromones as static modulators of arousal or valence, our findings highlight their dynamic role in shaping how odours are processed and categorised.

One limitation of this study is that the two odour pairings were trained at different times, with the octanal⁺/hexanal⁻ condition assessed in March-April and the hexanal⁺/octanal⁻ condition in May. Colony representation across the pheromone treatments (formic acid and water) was well balanced, but representation across odour pairings varied slightly due to fluctuations in forager availability across time. Colony identity was included as a random effect in all statistical models to account for potential variation in responsiveness or learning. Importantly, the learning curves observed in our control group closely match those reported by Perez et al. (2016), who used the same odour pairings under different temporal conditions. This consistency indicates that odour identity exerts a robust and replicable influence on learning performance, independent of seasonal variation. Nonetheless, future studies should aim to fully randomise odour pairings across time to eliminate any potential confounding effects of temporal structure.

Future research should explore whether similar cognitive effects occur in ecologically relevant settings, such as nestmate recognition or foraging, to assess the broader adaptive significance of alarm pheromone-induced cognitive modulation. More broadly, our findings provide the first evidence that an alarm pheromone can act asymmetrically on both learning and generalisation in ants, rather than merely influencing cognitive flexibility. This highlights a novel role for alarm pheromones as modulators of associative learning, actively reshaping stimulus discrimination and response patterns depending on context. Investigating whether other alarm signals similarly induce asymmetrical learning and generalisation effects in social insects or other taxa would deepen our understanding of the evolution of chemical communication and its integration with learning systems.

## Data Availability

The dataset and code supporting the findings of this study are publicly available in Figshare at https://doi.org/10.6084/m9.figshare.28677764.
